# Over-the-Counter Medicines, How to Avoid Causing Health Problems?

**DOI:** 10.3389/fphar.2012.00179

**Published:** 2012-10-08

**Authors:** Theophile Godfraind

**Affiliations:** ^1^Pharmacology, Université Catholique de LouvainBrussels, Belgium

Over-the-Counter medicines (OTC) can be bought either in drugstores or in pharmacies without a medical prescription in most countries in the world. Interestingly in some countries they are sold on stalls with food and spices in open-air markets. In the first case, there is guarantee on the chemical nature of the product, but in open-air markets, great uncertainties are usual. Like other products, they can be obtained from internet by a procedure, which looks more sophisticated than from an African open-air market, but doubts related to their chemical cleanliness might be similar. In the United States, the commercialization of any OTC medicine needs approval of the Food and Drug Administration, which decides whether it is safe enough to be sold over-the-counter. Furthermore, there are web sites issued from either NIH^1^ or FDA^2^ providing guidance to consumers. OTC drugs lists and books are easily accessible from Web search engines. Taking OTC has risks that are often not considered by buyer. Therefore in this field, education is a matter of public interest. OTCs are usually used for self-medication. A brief survey of literature showed that the prevalence of their use is variable when considering ages, sex, and supposed pathology. OTC pain relievers are the most common and may reach about 50% of patients in some polls (Wilcox et al., 2005).

Jean-Paul Giroud, the leading author of the book, is a renowned Clinical Pharmacologist from the French National Academy of Medicine in Paris. He did bring together a medical advisory board from wide-ranging specialties to review the book for accuracy. The purpose of the author was to provide appropriate information about OTC medicines by considering the following categories: those therapeutically efficient and not harmful, those devoid of therapeutic and toxic actions, those therapeutically efficient but inducing dangerous side effects and those without therapeutic interest but causing toxic reactions. According to J. P. Giroud there are about 4,000 OTC drugs available in France, but only 50% show some efficacy. The French official agency allowing commercialization of OTC drugs cannot guarantee the therapeutic index of those agents since the statistical power of clinical trials evaluating their action is often quite low. Therefore, Giroud scored the various preparations obtainable in France by a scale varying from 18 down to 0 on the basis of drug efficacy and safety, those parameters being semi-quantitative (Table 1).

**Table 1:** **Semi-quantitative scores of OTC medicines**.

**Score**	**OTC characteristic**
18–16	Only one active ingredient of good therapeutic efficiency and not harmful
15–12	Only one active ingredient of fair therapeutic efficiency and not harmful
11	Low therapeutic efficiency but not harmful
10	Therapeutic efficiency not demonstrated but not harmful
<10	Pharmacologically active but harmful
5–3	No therapeutic efficiency but harmful
2–0	Absence of pharmacological effect, should be withdrawn from the market

Subjects practicing self-medication and reading French language could use Giroud’s book as a support when they decide choosing any treatment. Indeed the first chapter is dealing with self-medication. It is assumed that this practice has advantages in case of minor health problems by preventing useless medical consultation and so saving money. However this might hide some symptoms indicators of a serious disease, an effect potentially deleterious for the patient. Consequently author enumerated 10 Commandments for reducing hazards. The most important are the following: avoiding drug accumulation, alcohol consumption, children and pregnant/feeding women self-medication, and uncontrolled use by old patients. He did not recommend phytotherapy because some preparations might be unsafe when containing added chemicals or even *per se* like Chinese herbs for weight control. In an easy-to-read section, he insisted on the properties of placebo and nocebo as well as on some pharmacokinetic aspects and the role of kidney and liver disease and of drug interactions in their modification. A major recommendation is that in case of medical consultation, the patient should inform the doctor on the practice of self-medication, not omitting to specify all preparations used.

The longer chapter (436 pages) deals with enumeration of symptoms and adult diseases presented in alphabetic order, which are linked to scores of OTC drugs used for obtaining their alleviation. For instance, for hair loss (alopecia), he described its origin and scored 12 on the preparations containing 2% minoxidil when noting that the drug is inactive in women. Symptoms and treatment of pediatric diseases (45 pages) are reported separately.

In conclusion, this authoritative book is as easy for use as a dictionary. It is full of advices for staying away from risk of drug intoxication, so it contributes to securing self-medication. By avoiding technical terminology, it also gives to layman useful information on most common diseases. It constitutes an educational intervention directed toward patients. Its originality is lying in OTC medicines scoring, which offers a convincing warning for not taking inappropriate and potentially dangerous agents, so protecting users that are generally unaware of the prospect of adverse side effects.

## Footnotes

^1^http://www.nlm.nih.gov/medlineplus/overthecountermedicines.html

^2^http://www.fda.gov/drugs/developmentapprovalprocess/howdrugsaredevelopedandapproved/approvalapplications/over-the-counterdrugs/default.htm
